# 3D printing in surgical simulation: emphasized importance in the COVID-19 pandemic era

**DOI:** 10.2217/3dp-2021-0009

**Published:** 2021-05-10

**Authors:** Ross Michaels, Chelsey A Witsberger, Allison R Powell, Krishna Koka, Katheryn Cohen, Zahra Nourmohammadi, Glen E Green, David A Zopf

**Affiliations:** 1 ^1^Medical School, University of Michigan, Ann Arbor, MI 48109, USA; 2 ^2^Medical School, Indiana University, Indianapolis, IN 46202, USA; 3 ^3^Department of Biomedical Engineering, University of Michigan, Ann Arbor, MI 48109, USA; 4 ^4^Washington University in St Louis, St Louis, MO 63130, USA; 5 ^5^Department of Otolaryngology – Head & Neck Surgery, University of Michigan, Ann Arbor, MI 48109, USA

**Keywords:** 3D printing, 3D visualization, additive manufacturing, anatomical training, CAD/CAM, surgery, surgical planning, surgical training

## Brief review of 3D printing in surgical simulation

Historically, surgical training was an apprenticeship model of see one, do one, teach one. However, a proficiency-based training approach has become increasingly implemented for assessing surgical skills with performance scores used as benchmarks to track trainee proficiency [1]. Surgical simulators are starting to be utilized more to assess proficiency in trainees on certain procedures with many residency programs having simulation as a piece of their training curriculum. Today, simulation in surgical training takes many forms. Live animals and cadavers are often implemented since these simulators can simulate operating on realistic tissue and on human anatomy respectively. There are also basic simulators that are models that simulate a component of an operation such as suturing or knot-tying. These help trainees practice certain surgical skills necessary for completing a procedure. Some of these simulators have become more complex and simulate several steps or even an entire procedure such as joint replacements and fixating fractures [1].

With the increased availability in 3D printing technology and a push toward personalized medicine, 3D printing research has exponentially increased in recent years and has been an area of investigation for the development of surgical simulators [2]. Using a 3D printer to construct models for simulation leads to vast opportunity to customize the simulator while significantly reducing cost. Prior to the advent of 3D printing and additive manufacturing, computed tomography (CT) data were used to construct anatomic models using subtractive manufacturing with the first model made in 1979 [3]. Commercial 3D printers became available in the 1980s and were introduced into the medical field in 1994 [4]. Currently, 3D printing has several surgical applications including anatomic models for surgical planning, simulation and education; implants and prostheses; and surgical guides [3].

## Simulators role in surgical education

In the appropriate application, 3D-printed surgical simulators can have many advantages over other forms of simulation. Some examples of other simulation modalities currently being used include cadaveric simulations, animal model simulations, repurposing common materials and virtual reality (VR) training. Each modality has potential strengths and weaknesses as discussed below.

Cadaveric simulations are used due to the conservation of complex anatomy, which is difficult to achieve with any other form of simulation. Cadaveric simulation offers excellent haptic feedback and is useful for developing muscle memory in rehearsal of procedures. It should be noted that, depending on the postmortem treatment of the cadaver, tissue mechanics and responsiveness can be variable. The disadvantages of cadaveric simulation are that it requires donor bodies to be subject to postmortem manipulation and donors have been on the decline in recent years [5]. Cadaveric simulation is expensive, as it requires high-cost dissection labs and storage facilities. Cadaveric simulation is also highly regulated and the bodies are perishable [4]. They can also only display the anatomy present at the time of death and typically relay normal anatomy, not acquired or congenital pathology.

Animal models are exceptional simulators for cross-species shared anatomy. Animal models allow for real-time procedural management training when conducting procedures in anesthetized animals [2]. Animal models also boast excellent tissue mechanics and resultant haptic feedback. However, animal models are again expensive, they require high-cost dissection labs and are often inaccessible, highly regulated and perishable. While there is some cross-species anatomy conservation, there is also major variation that limits the scope of animal model simulation. There is also cultural and moral conflict of the sacrifice of animals for educational purposes. This conflict is more pronounced among people who hold animals as sacred due to religious or personal beliefs. Also, in the setting of the COVID-19 pandemic, many procedures using animal models have been put on hold.

Repurposed common materials are very common in surgical education. An example would be using a banana as a suturing board or using a bar of soap as practice for carving cartilage. Strengths of repurposing include the ready availability of materials and relatively low costs of this method. This method can also be used independently at home or in conjunction with virtual learning. Often, it is difficult to find common materials that simulate anatomical structures or possess the correct tissue mechanical properties [6].

Virtual reality (VR) simulators are increasing in popularity, but they lack rigorous peer review [7]. VR offers the ability to complete high volumes of simulated procedures with a wide variety of pathologies, postures and morphologies without large laboratory spaces or physical materials [8]. The virtual nature allows for reproducible, standardized practice, which is excellent for promoting recall and also for assessing learner’s skill progression. VR allows for transition from CT/MRI data to virtual models. VR and augmented reality (AR) also lack crucial haptic feedback cues that are a cornerstone of surgical education. Many learners can also find the equipment cumbersome and unintuitive. Both VR and AR remain costly when compared with other simulator modalities but with advancement in technology and the commercialization of this technology, cost will likely come down in the future [9].

3D-printed surgical simulators have a variety of notable strengths. 3D printing allows for rapid design and manufacturing of simulators in a cost-effective manner. As electronic files can be shared, using 3D printing to manufacture simulators, enables broad global distribution since simulators can be readily made anywhere with access to a 3D printer. Due to the customizability of using a 3D printer, 3D-printed simulators can easily be designed to simulate specific patient pathology using image processing of patient CT or MRI files. As a result, physicians can accurately simulate a procedure accounting for anatomic variants between patients. 3D-printed simulators are also largely nonperishable, making them archivable, which affords many advantages such as tracking resident skill acquisition over time. The nonperishable nature of these simulators also allows for easy distribution and use in remote locations, an advantage that has become readily apparent in the setting of the COVID-19 pandemic [10].

Simulator consumable material cost can be very low. Two examples are our costal cartilage simulator at $0.60 and cleft lip simulator at $4.59 [11,12]. The most common raw materials used in our lab are polylactic acid (PLA) costing $0.025/g and moldable silicone costing approximately $110/gal [13,14].

However, 3D-printed simulators are not without their own disadvantages. The first is that they require an initial investment in a 3D printer. Cost is coming down with time, and an alternative approach is collaboration with a group already active in this space. Second, 3D printing requires access to and knowledge of computer-aided design (CAD) software and basic 3D printing software in order to create new simulators and successfully print them. Software can be expensive based on complexity and feature array. At last, 3D printing can be limited by printer precision and material characteristics of the printed materials. These limitations should always be considered prior to choosing 3D printing as a simulation modality.

## Illustrative examples of 3D-printed surgical simulators

Our lab at the University of Michigan under the direction of two otolaryngologist, head and neck surgeons, is continually expanding. Our library of surgical simulators initially focused on the head and neck surgical fields has expanded through collaborations with experts in the fields of oral maxillofacial surgery, pediatric surgery, cardiothoracic surgery and nonmedical arenas such as our institution’s School of Music. Examples of high fidelity simulators we have developed include airway reconstruction, cleft palates, cleft lips, ear reconstruction and facial flaps, with the number of procedures having grown such that we are now organizing 3D-printed simulator dissection courses [15].

Our simulators are developed using the following pathway. We import de-identified patient data from a patient with a given pathology to Materialise Mimics software (Materialise, Inc., MI, USA). From there we are able to create a CAD mask of the given anatomy. The 3D masks are then used to render a 3D object which is imported into Materialise 3Matic software (Materialise, Inc.). Within 3Matic, all objects go through postrendering processing to remove unintended artifact due to noise within the imaging file or software limitations within the mask creation. This process normally involves some computer aided cutting, hole filling and smoothing.

Objects are then divided into two general categories: osseus/solid components and soft tissue components. Negative molds filled with silicone are used to create soft tissue objects. These molds range in complexity from having one solid part to having multiple integrated pieces – which can allow for molded pieces to be removed after setting. STL files from 3Matic are used to print solid objects and molds via additive manufacturing. We use both fused filament fabrication (FFF) and also stereolithography 3D printers in our lab. FFF printers are used when not prohibited by precision limitations due to low material costs. We currently use PLA for most of our FFF applications. When higher precision is necessary, stereolithography is utilized. For our soft tissue parts, we have found that most tissues can be replicated by using different combinations of silicones ranging in stiffness from Shore 10–50 with addition of silicone additives or modifiers.

Following fabrication our models are put through a process of expert validation in which experts experienced in the correction of a given pathology perform corrective procedures on the simulators. Experts then rate them based on themes such as anatomical accuracy, simulator realism and surgical education value. Our lab is not unique in its methods as 3D-printed surgical simulators have been created in many other surgical fields across the world. The one barrier to implementation of our lab’s above, simplified method is the cost of computer software (image processing and CAD software). Open-source software and open-source hardware communities are slowly decreasing this barrier through increasing accessibility.

## Simulation in the pandemic era

On 10 March 2020, our state entered a state of emergency due to the rapidly spreading COVID-19 virus. In order to protect life, property and conserve personal protective equipment (PPE), the state Executive Order #2020-17 placed a restriction on nonessential medical and dental procedures in the state [16]. Similar cessation was seen nationally and internationally during the initial COVID-19 response. This stopped elective surgery cases around the state, causing a dramatic decrease in the number of cases available for the education of surgical residents. Compounding this problem, in nonelective cases, the number of providers was often restricted to limit exposure during aerosolizing procedures and again to preserve PPE. In a survey of 1102 general surgery residents during the initial COVID-19 response, 42.3% of general surgery respondents felt they would not meet the traditional Accredited Council for Graduate Medical Education (ACGME) case requirements for graduation with the changes made to their program as a result of COVID-19 [17]. The reduction in case load, with no foreseeable end in sight at the beginning of the pandemic, was detrimental to the education of surgical residents and the development of critical surgical skills during their relatively short period of training. Other educational opportunities such as surgical symposiums, surgical conferences and cadaveric dissection courses were also put on hold or held virtually during this time.

Surgical simulation offers a robust, viable solution to addressing the reduction in training opportunities. Social distancing principles can readily be maintained. We believe that 3D printing remains a predominant method for creation of these simulators in the correct application. With high fidelity surgical simulators that can be rapidly 3D printed and a virtual curriculum, these residents could learn valuable surgical skills in remote settings. A successful example of this is the ‘take home box’ simulation kits which included a 3D-printed simulator and all necessary dissection tools conveniently packaged for pick-up [6]. Patel *et al.* outline the objective evaluation of their implemented anesthesiology ‘telesimulation’ [18]. Examples like these in combination with the rapid advancement in teleconferencing software could allow for a full cohort of students to learn and rehearse steps in surgical procedures, such as the repair of a pediatric cleft palate, while under the virtual supervision of an attending physician who can offer real time feedback just as they would in the operating room.

## Future perspective

In early 2020, many surgical residency programs were dramatically impacted by the sudden changes required by COVID-19. In order to be prepared for new waves of the coronavirus or other unforeseen events that may impact Graduate Medical Education, it is evident that surgical education requires diversification of the educational opportunities afforded to surgical residents. 3D printing of surgical simulators offers opportunities for surgical education of the future. COVID-19 further highlighted this opportunity and our team continues to explore its broad value in domestic GME and international educational initiatives.

One, the development of new, 3D-printed simulators is necessary to simulate a higher degree of different surgical specialties. When possible, academic centers may find it valuable to partner with the open source community for the development of these simulators. Two, educational curriculums need to be designed with robust social distancing principles in mind. Curriculums developed for nonpandemic era education need to be re-evaluated to have socially-distanced alternatives that can be rapidly deployed. Three, whenever possible, we must pursue national and international collaboration to increase accessibility to surgical training opportunities. In following these steps, we believe that the surgical community will be more readily prepared in the event of future closures but also will be vastly benefited by a new, expanding and versatile surgical education solution.
